# Metamorphopsia Associated with Branch Retinal Vein Occlusion

**DOI:** 10.1371/journal.pone.0153817

**Published:** 2016-04-28

**Authors:** Koichiro Manabe, Akitaka Tsujikawa, Rie Osaka, Yuki Nakano, Tomoyoshi Fujita, Chieko Shiragami, Kazuyuki Hirooka, Akihito Uji, Yuki Muraoka

**Affiliations:** 1 Department of Ophthalmology, Kagawa University Faculty of Medicine, Miki, Japan; 2 Department of Ophthalmology and Visual Sciences, Kyoto University Graduate School of Medicine, Kyoto, Japan; University of Florence, ITALY

## Abstract

**Purpose:**

To apply M-CHARTS for quantitative measurements of metamorphopsia in eyes with acute branch retinal vein occlusion (BRVO) and to elucidate the pathomorphology that causes metamorphopsia.

**Methods:**

This prospective study consisted of 42 consecutive patients (42 eyes) with acute BRVO. Both at baseline and one month after treatment with ranibizumab, metamorphopsia was measured with M-CHARTS, and the retinal morphological changes were examined with optical coherence tomography.

**Results:**

At baseline, metamorphopsia was detected in the vertical and/or horizontal directions in 29 (69.0%) eyes; the mean vertical and horizontal scores were 0.59 ± 0.57 and 0.52 ± 0.67, respectively. The maximum inner retinal thickness showed no association with the M-CHARTS score, but the M-CHARTS score was correlated with the total foveal thickness (r = 0.43, p = 0.004), the height of serous retinal detachment (r = 0.31, p = 0.047), and the maximum outer retinal thickness (r = 0.36, p = 0.020). One month after treatment, both the inner and outer retinal thickness substantially decreased. However, metamorphopsia persisted in 26 (89.7%) of 29 eyes. The posttreatment M-CHARTS score was not correlated with any posttreatment morphological parameters. However, the posttreatment M-CHARTS score was weakly correlated with the baseline total foveal thickness (r = 0.35. p = 0.024) and closely correlated with the baseline M-CHARTS score (r = 0.78, p < 0.001).

**Conclusions:**

Metamorphopsia associated with acute BRVO was quantified using M-CHARTS. Initial microstructural changes in the outer retina from acute BRVO may primarily account for the metamorphopsia.

## Introduction

Macular edema (ME) is one of the main causes of the decrease in visual acuity (VA) associated with acute branch retinal vein occlusion (BRVO) [1]. The visual prognosis of BRVO has been substantially improved since the introduction of anti-vascular endothelial growth factor agents [2, 3]. However, some patients suffer from a decreased quality of vision due to symptomatic metamorphopsia, even after the complete reduction of ME. Recent advancements in ophthalmic instruments have improved our understanding of the pathophysiology of retinal complications associated with BRVO and their correlations with subjective symptoms [4]. However, our understanding of metamorphopsia caused by BRVO remains limited due to the lack of methodologies to quantify the degree of metamorphopsia [5–10]. In addition, BRVO, which is caused by a circulatory disturbance within the inner retina, shows various features, such as retinal swelling, retinal cystoid spaces, retinal hemorrhage, non-perfusion area, and serous retinal detachment, and these morphological changes extend from the inner retina to the subretinal spaces [1]. Such complexity in the pathologic features makes it difficult to elucidate the pathogenesis of metamorphopsia associated with acute BRVO.

M-CHARTS, developed by Matsumoto et al.[11], allows us to quantitatively evaluate the degree of metamorphopsia. M-CHARTS consists of a series of 19 dotted line tests in which the intervals of each dot range from 0.2° to 2.0°. In patients with metamorphopsia, a dotted line with a small interval is often recognized as distorted. As the dot interval increases, the distortion of the line decreases. With M-CHARTS, metamorphopsia is quantified as the minimum interval at which no visual distortion is present. To date, quantitative evaluations with M-CHARTS have been applied in epiretinal membrane [11–21], rhegmatogenous retinal detachment [22], age-related macular degeneration [23, 24], diabetic macular edema [25], macular hole [26, 27], and central serous chorioretinopathy [28, 29]. Based on the use of M-CHARTS for epiretinal membrane, it has been reported that the severity of metamorphopsia is related to the thickness of the inner nuclear layer measured with optical coherence tomography (OCT) [15, 21, 30] or foveal microfolds visualized using adaptive optics-scanning laser ophthalmoscopy [13].

However, few investigators have quantitatively evaluated metamorphopsia associated with BRVO; using M-CHARTS, Nakagawa et al.[6], studied metamorphopsia in 12 eyes with BRVO, and Achiron et al.[5] detected metamorphopsia in 4 eyes with retinal vein occlusion. To the best of our knowledge, no previous reports have described the retinal pathomorphology that is involved in the metamorphopsia associated with BRVO. Therefore, the purposes of the current study were (1) to perform quantitative measurements of metamorphopsia with M-CHARTS in eyes with acute BRVO in order to determine the prevalence and severity of metamorphopsia and (2) to compare the M-CHARTS score with the retinal morphology measured using OCT in order to elucidate the pathomorphology that causes metamorphopsia.

## Patients and Methods

This study was approved by the Ethics Committee at Kagawa University Faculty of Medicine and conducted in accordance with the tenets of the Declaration of Helsinki. Written informed consent was obtained from each subject before any study procedures or examinations were performed.

### Patients

This prospective study consisted of 42 consecutive patients (42 eyes) with ME associated with acute BRVO who were examined and treated at the Department of Ophthalmology of Kagawa University Hospital between September 2014 and September 2015.

The inclusion criteria of this study were (1) symptomatic BRVO, in which retinal hemorrhage and retinal edema involved the macula, (2) foveal thickness of greater than 250 μm at the initial visit as measured by OCT, and (3) a duration of symptoms prior to the initial examination of less than 3 months. The diagnosis of BRVO was based on fundus examinations and fluorescein angiography findings determined by two retina specialists (KM, AT). Eyes with central retinal vein occlusion or hemi-central retinal vein occlusion were not included in the current study. Eyes with co-existing ocular disease (*i*.*e*., age-related macular degeneration, retinitis pigmentosa, diabetic retinopathy, retinal macroaneurysm, or senile cataract that resulted in poor image quality), and eyes that had any history of interventions for ME before inclusion in the study were excluded.

### Schedule of evaluation

At the initial visit, the medical history was obtained from each patient. Each patient underwent a comprehensive ophthalmologic examination, including measurement of the best-corrected VA using the Landolt chart and the degree of metamorphopsia by M-CHARTS (Inami, Tokyo, Japan), determination of intraocular pressure, indirect ophthalmoscopy, slit-lamp biomicroscopy with a noncontact lens, OCT examinations (Spectralis HRA+OCT; Heidelberg Engineering, Heidelberg, Germany), and fluorescein angiography (Optos 200Tx imaging system, Optos PLC, Dunfermline, United Kingdom).

Each patient was treated with an intravitreal injection of ranibizumab (Lucentis; Novartis Pharma, Tokyo, Japan) for ME. In order to evaluate the retinal morphology and visual function during the recovery from ME, each patient was scheduled for reevaluation of the retinal morphology and visual function one month after the initial injection. One month after the treatment, all eyes showed a marked reduction of ME and frequently achieved an improvement of visual symptoms and VA. Each patient underwent a complete ophthalmologic examination, including measurement of the VA and M-CHARTS score, slit-lamp biomicroscopy, indirect fundus ophthalmoscopy, and OCT examination. Fluorescein angiography was performed as necessary.

Each patient was examined at our clinic every month. Thereafter, most eyes showed the indeterminate recurrence of ME. Additional injections were performed when ME and/or serous retinal detachment was evident at the fovea on OCT examination.

### Metamorphopsia evaluation

**Fig 1:**
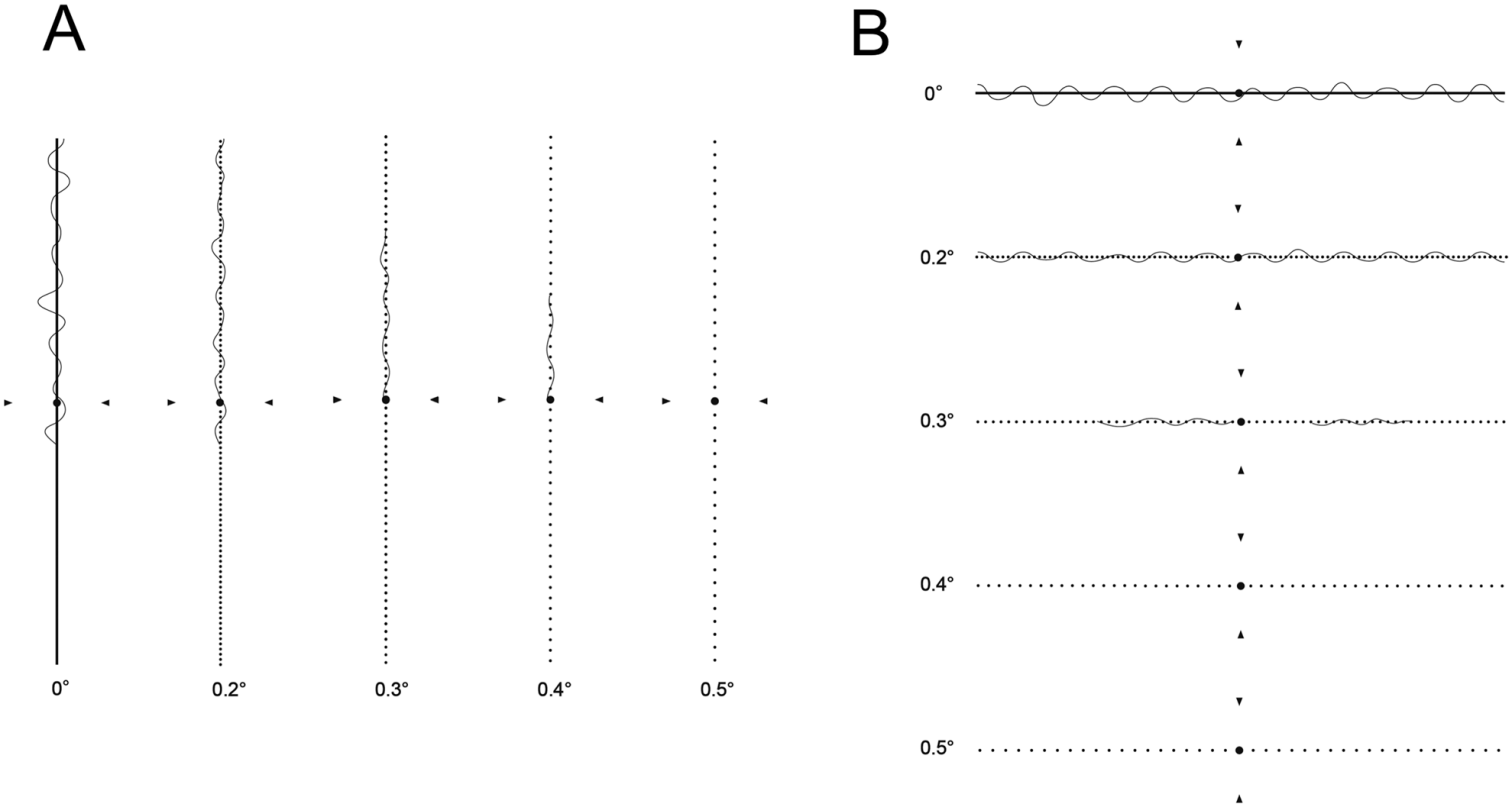
Quantitative measurement of metamorphopsia using M-CHARTS in eyes with acute branch retinal vein occlusion. First, a chart with a solid line was presented at a distance of 30 cm. Thereafter, charts with dotted lines with incrementally larger spacing were presented. When the patient recognizes the presented line as being straight, the visual angle of that line is taken as the metamorphopsia degree. The vertical and horizontal score was measured, and the higher score was used as the M-CHARTS score. In this eye, the vertical (**A**) and horizontal (**B**) metamorphopsia were 0.5 and 0.4. The M-CHARTS score of this eye is 0.5.

M-CHARTS is commercially available to quantify the degree of metamorphopsia. The principal of M-CHARTS has been described in detail previously (Fig 1) [11]. In brief, M-CHARTS is composed of a series of 19 dotted line tests. In each chart, the intervals of each dot range from 0.2° to 2.0°. A fixation point is printed in the center of each line, measuring 0.3° of the visual angle. First, an examiner presents a chart with a solid line at a distance of 30 cm under the correction of the refraction, followed by charts with dotted lines of incrementally increasing spacing. For each chart, the patient is asked to state whether the presented line is distorted or not. As the visual angle increases, the degree of metamorphopsia decreases. When the patient recognizes the presented line as being straight, the visual angle of that line is taken as the degree of metamorphopsia. M-CHARTS were presented to the patient in a vertical direction and then in a horizontal direction. The vertical and horizontal scores were measured, and the higher score was used as the M-CHARTS score for the eye.

### Measurement of retinal structural changes with optical coherence tomography

Morphologic evaluations and quantitative measurements of ME associated with BRVO were performed by OCT. The entire macular area was examined with sequential OCT sectioning to detect any serous retinal detachment or cystoid spaces. Quantitative measurements were performed using a vertical section acquired through the foveal center because the BRVO-affected retina was mainly located on either the upper hemisphere or the lower hemisphere of the retina. In the current study, the thickness of the inner retina was defined as the vertical distance between the vitreoretinal interface and the outer surface of the inner nuclear layer. The thickness of the outer retina was defined as the vertical distance between the outer surface of the inner nuclear layer and the inner surface of the retinal pigment epithelium. The total retinal thickness was defined as the distance between the vitreoretinal interface and the inner surface of the retinal pigment epithelium.

**Fig 2:**
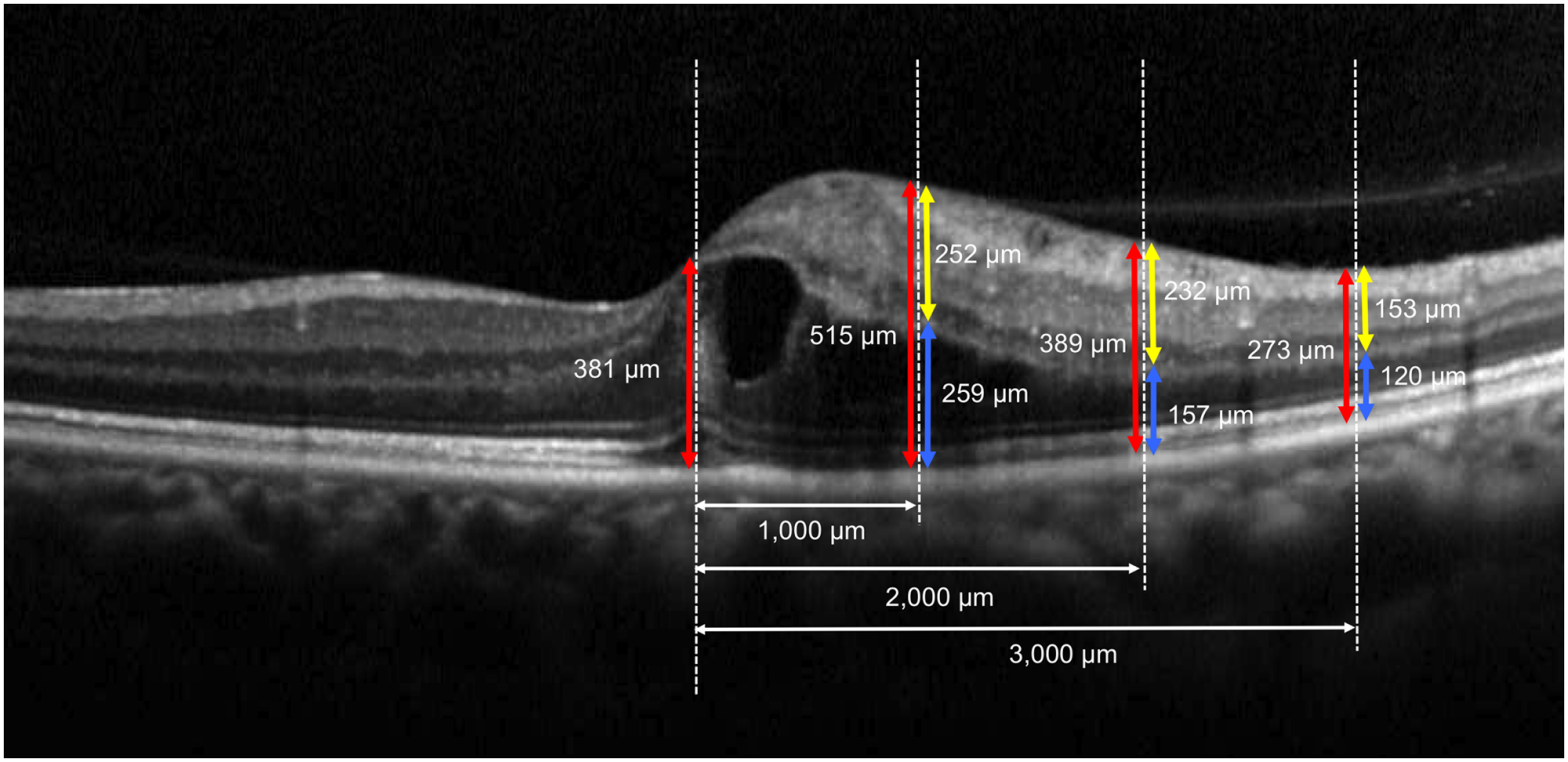
Quantitative measurements of retinal morphological changes associated with acute branch retinal vein occlusion with optical coherence tomography. On the vertical section through the foveal center, the inner (yellow arrows), outer (blue arrows), and total (red arrows) retinal thickness was measured at 1 mm, 2 mm, and 3 mm from the center of the fovea toward the affected side of the retina, respectively. The maximum thickness of the inner, outer, or total retina was determined as the largest among the three measurements.

On the vertical section through the foveal center, the inner, outer, and total retinal thickness were measured at 1 mm, 2 mm, and 3 mm from the foveal center on the affected retinal side, respectively (Fig 2). The maximum thickness of the inner, outer, or total retina was defined as the maximum value among the three measurements (Fig 2). The thickness of the serous retinal detachment was measured manually at the largest point, which was frequently at the fovea [31]. These measurements were performed at baseline and one month after the initial treatment by one grader (KM) in a masked fashion.

### Statistical analysis

Statistical analysis was performed using SPSS, version 21.0.0 (IBM Japan, Tokyo, Japan). All values are presented as the means ± standard deviation. The best-corrected VA was converted to a logarithm of the minimum angle of resolution (logMAR) equivalent for statistical analysis. The M-CHARTS score was considered to be changed when the change in score was greater than 0.1 [11]. Comparisons between baseline and posttreatment values were performed using the paired *t*-test. Bivariate relationships were analyzed using the Pearson’s correlation coefficient to evaluate the correlation between each measurement value and the M-CHARTS score. A value of p < 0.05 was considered statistically significant.

## Results

Table 1 shows the baseline measurements of all patients eligible in this study. At baseline, all eyes showed visual disturbance associated with acute BRVO; the mean VA was 0.33 ± 0.31, and the mean total foveal thickness was 467.2 ± 191.5 μm. The maximum inner, outer, and total retinal thickness was 312.6 ± 90.4 μm, 294.5 ± 114.3 μm, and 588.3 ± 163.9 μm, respectively. Of 42 eyes, 21 (50.0%) showed serous retinal detachment at the fovea.

**Table 1:** Baseline Characteristics of Eyes with Acute Branch Retinal Vein Occlusion.

Age, years	69.0 ± 11.4
Gender, women/men	20/22
Duration of symptom until examination, weeks	5.5 ± 5.2
Visual acuity, logMAR	0.33 ± 0.31
Total foveal thickness, μm	467.2 ± 191.5
Thickness of serous retinal detachment, μm	149.0 ± 95.7
Maximum of total retinal thickness, μm	588.3 ± 163.9
Maximum of inner retinal thickness, μm	312.6 ± 90.4
Maximum of outer retinal thickness, μm	294.5 ±114.3

All values are presented as the mean ± standard deviation.

logMAR, logarithm of the minimum angle of resolution.

Metamorphopsia was quantified using M-CHARTS. Of 42 eyes, no metamorphopsia was detected in 13 (31.0%) eyes. Metamorphopsia was detected in the vertical and/or horizontal directions in 29 (69.0%) eyes. The mean vertical and horizontal scores were 0.59 ± 0.57 and 0.52 ± 0.67, respectively (Table 2). The vertical score was slightly greater than the horizontal score, although the difference was not significant (p = 0.070).

**Table 2:** Baseline Score of Metamorphopsia Measured with M-CHARTS.

Vertical score	0.59 ± 0.57
Horizontal score	0.52 ± 0.67
Higher score	0.68 ± 0.67

Higher score is determined from the vertical and horizontal scores.

**Fig 3:**
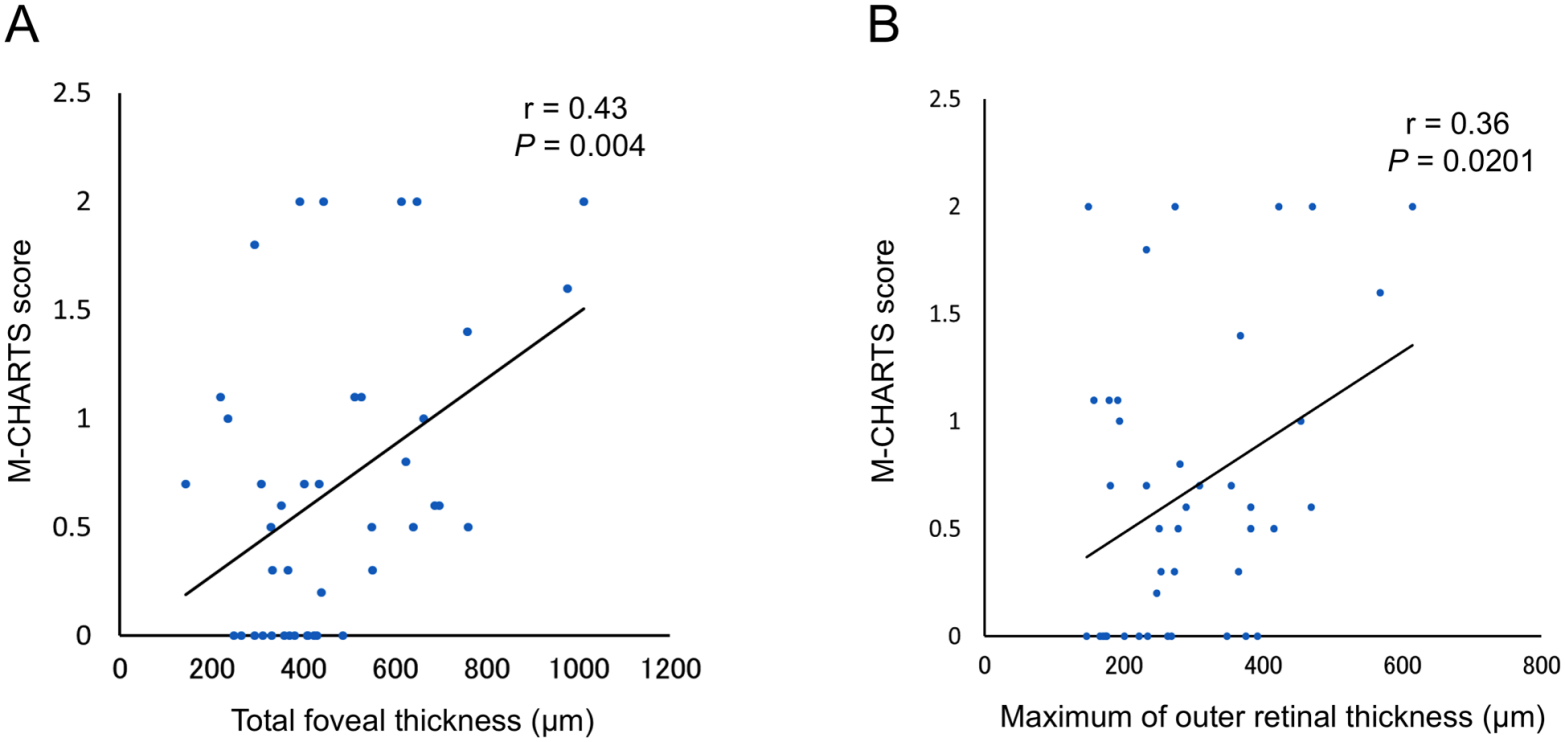
Association between the M-CHARTS score and the total foveal thickness (A) and the maximum outer retinal thickness (B) in the macular area affected by acute branch retinal vein occlusion.

The higher score of the vertical and horizontal scores was used as the M-CHARTS score. Table 3 shows the association of the baseline M-CHARTS score and the baseline morphological parameters with OCT. While the VA showed no association with the M-CHARTS score, the total foveal thickness was associated with the M-CHARTS score (r = 0.43, p = 0.004). The M-CHARTS score had no association with the maximum inner retinal thickness (r = 0.10, p = 0.527), but it was correlated with the thickness of the serous retinal detachment (r = 0.31, p = 0.047) and with the maximum outer retinal thickness (r = 0.36, p = 0.020; Fig 3). Metamorphopsia seemed to be mainly associated with the morphological changes of the outer aspect of the retina.

**Table 3:** Association between Baseline M-CHARTS Score and Baseline Pathomorphological Parameters of Eyes with Acute Branch Retinal Vein Occlusion.

	r	p-value
Age	0.15	0.342
Visual acuity (logMAR)	0.19	0.219
Total foveal thickness	0.43	0.004
Thickness of serous retinal detachment	0.31	0.047
Maximum of total retinal thickness	0.25	0.116
Maximum of inner retinal thickness	0.10	0.527
Maximum of outer retinal thickness	0.36	0.020

logMAR, logarithm of the minimum angle of resolution.

M-CHARTS Score is the higher score of the vertical and horizontal scores of M-CHARTS.

**Fig 4:**
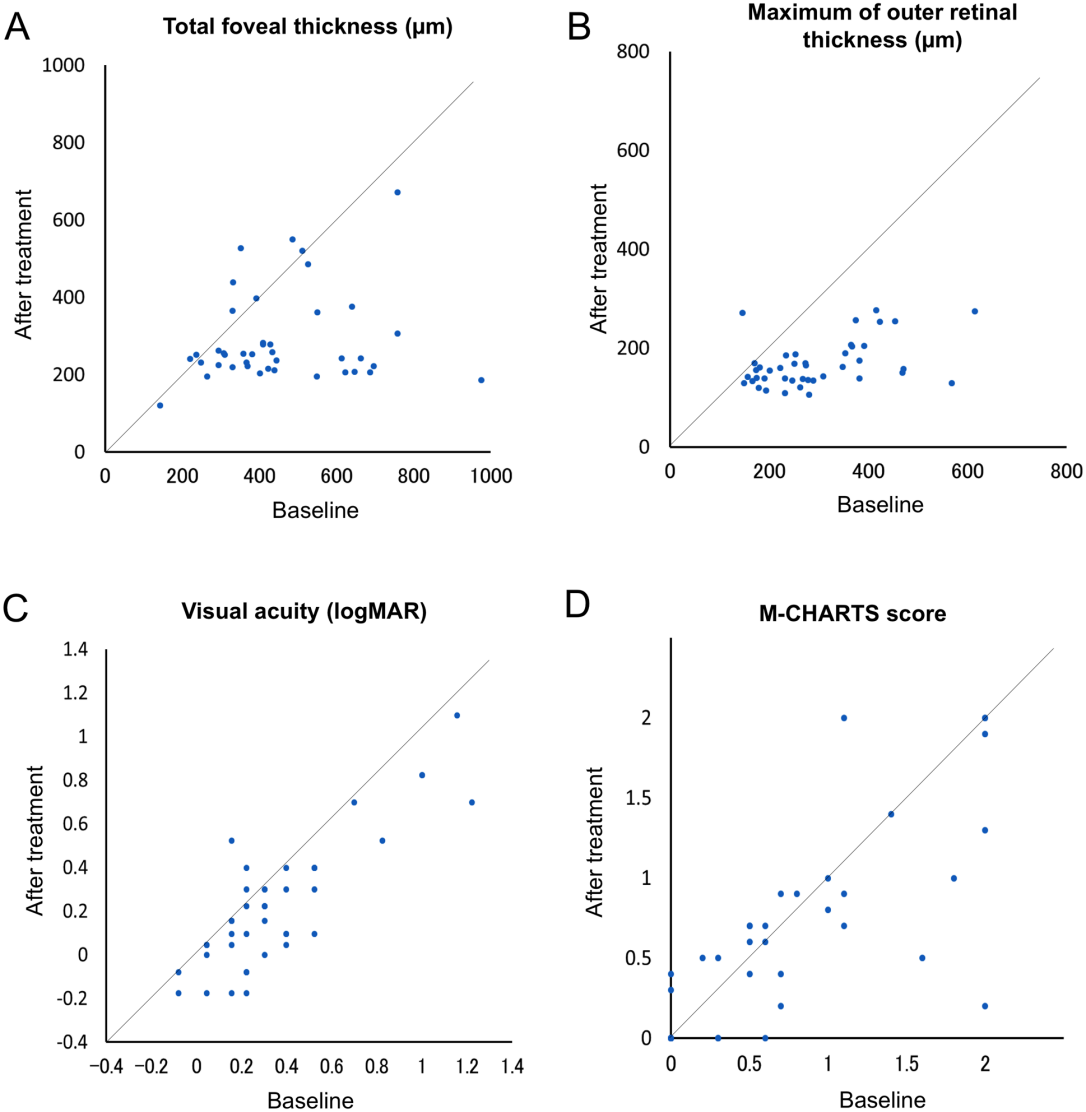
Correlations between the baseline and the posttreatment total foveal thickness (A), maximum outer retinal thickness (B), visual acuity (C), and M-CHARTS score (D) in eyes with acute branch retinal vein occlusion that were treated with an intravitreal injection of ranibizumab. One month after the initial treatment, the maximum outer retinal thickness and total foveal thickness and was significantly decreased (both p < 0.001). The visual acuity in logMAR was also significantly improved (p < 0.001). The improvement in the mean M-CHARTS score was not significant (p = 0.050), and 28 eyes still had metamorphopsia.

**Fig 5:**
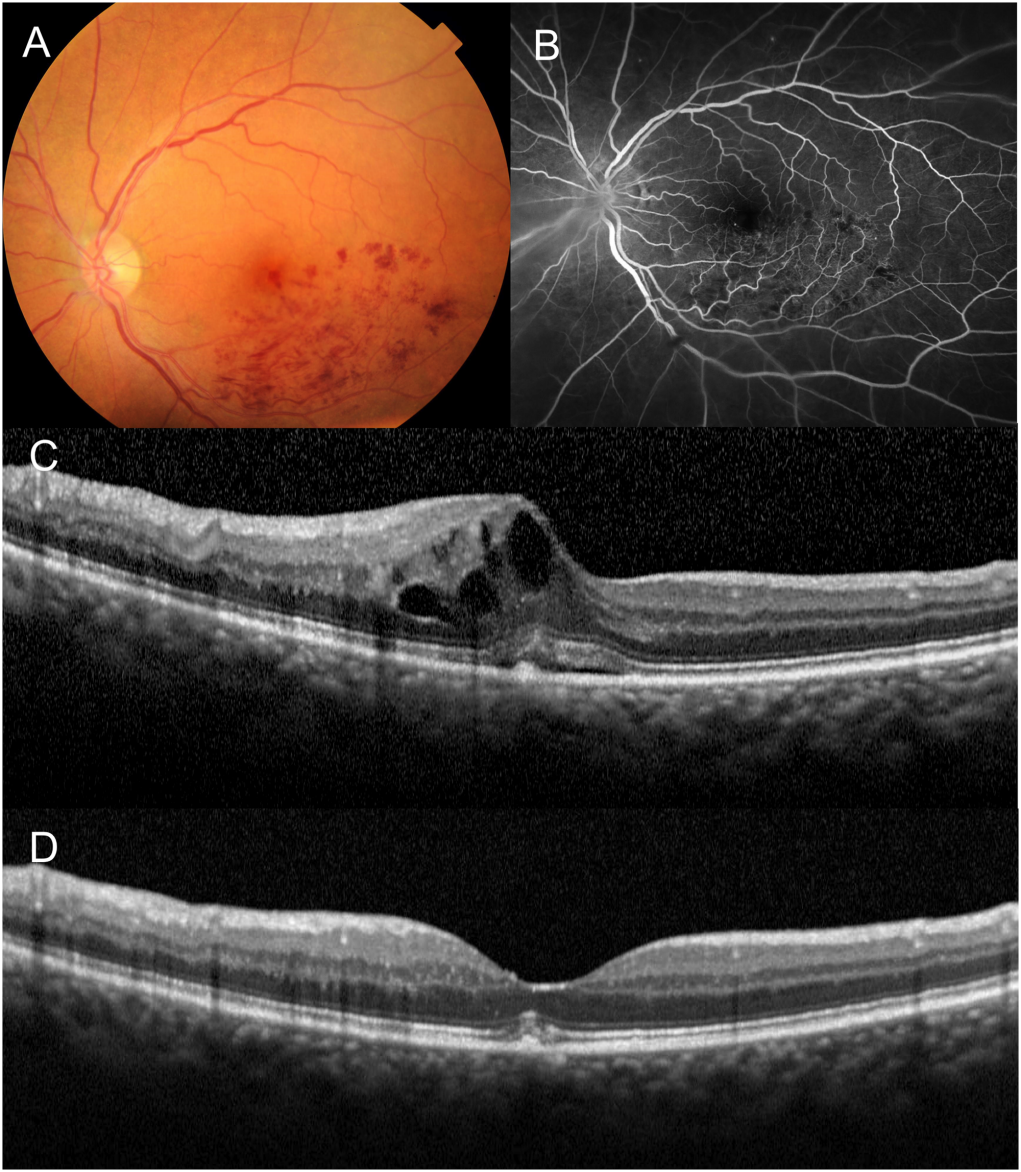
Persistent metamorphopsia after the resolution of macular edema associated with acute branch retinal vein occlusion. A 74-year-old woman had visual disturbance due to acute BRVO in the left eye. (**A**) Fundus photograph. (**B**) Fluorescein angiogram. (**C**) The vertical section of an optical coherence tomography (OCT) scan through the foveal center shows the foveal cystoid spaces and retinal thickening in the affected retina. The visual acuity of the left eye was 0.30 logMAR. The vertical and horizontal M-CHARTS scores were 1.0 and 0.7, respectively. The eye was treated with an intravitreal injection of ranibizumab. (**D**) One month after the injection, a vertical OCT section shows the complete absorption of the macular edema. The visual acuity was improved to 0.15 logMAR. However, the vertical and horizontal M-CHARTS scores were still both 0.8.

One month after the initial treatment, most eyes showed a marked reduction of ME (Table 4). Both the inner and outer retinal thickness were significantly decreased, not only at the fovea, but also at the parafoveal area (p < 0.001). The mean VA was improved from 0.33 ± 0.31 to 0.23 ± 0.29 (p < 0.001). However, metamorphopsia persisted in most eyes, although there was some improvement (Fig 4). Of 29 eyes that had metamorphopsia at baseline, metamorphopsia was completely eliminated in only three (10.3%) eyes, and it persisted in 26 (89.7%) eyes (Fig 5). In addition, two eyes developed metamorphopsia after treatment. Of 42 eyes, the M-CHARTS score was decreased in 13 (31.0%) eyes and increased in 8 (19.0%) eyes after the treatment. The change in the M-CHARTS score after the treatment was significantly correlated with the baseline score (r = -0.48, p = 0.001). The mean M-CHARTS score was decreased with treatment, but the improvement was not significant (p = 0.050).

**Table 4:** Measurement Values Associated with Acute Branch Retinal Vein Occlusion at Baseline and One Month after Initial Treatment with Ranibizumab.

	Baseline	One Month	p-value
Visual acuity, logMAR	0.33 ± 0.31	0.23 ± 0.29	< 0.001
Total foveal thickness, μm	467.2 ± 191.5	287.8 ± 115.8	< 0.001
Thickness of serous retinal detachment, μm	149.0 ± 95.7	55.0 ± 39.8	0.069
Maximum of total retinal thickness, μm	588.3 ± 163.9	437.3 ± 87.4	< 0.001
Maximum of inner retinal thickness, μm	312.6 ± 90.4	244.9 ± 54.8	< 0.001
Maximum of outer retinal thickness, μm	294.5 ±114.3	204.1 ± 60.5	< 0.001
M-CHARTS			
Vertical score	0.59 ± 0.57	0.50 ± 0.51	0.020
Horizontal score	0.52 ± 0.67	0.43 ± 0.54	0.150
Higher score	0.68 ± 0.67	0.57 ± 0.60	0.050

logMAR, logarithm of the minimum angle of resolution.

M-CHARTS Score is the higher score of the vertical and horizontal scores of M-CHARTS.

Table 5 shows the association between the posttreatment M-CHARTS score and the other posttreatment measurement values. Of 42 patients, 28 eyes had metamorphopsia after the treatment. No posttreatment factors had an association with the posttreatment M-CHARTS score. Table 6 shows the association between the posttreatment M-CHARTS score and the baseline measurement values. The posttreatment M-CHARTS score was weakly correlated with the total foveal thickness (r = 0.35, p < 0.024) and closely correlated with the M-CHARTS at baseline (r = 0.78, p < 0.001).

**Table 5:** Association between Posttreatment M-CHARTS Score and Other Measurement Values at One Month after Initial Treatment with Ranibizumab.

	r	p-value
Age	0.20	0.215
Visual acuity (logMAR)	0.28	0.072
Total foveal thickness	0.10	0.389
Thickness of serous retinal detachment	-0.14	0.389
Maximum of total retinal thickness	-0.07	0.646
Maximum of inner retinal thickness	0.12	0.445
Maximum of outer retinal thickness	-0.20	0.212

logMAR, logarithm of the minimum angle of resolution.

Measurement of M-CHARTS was performed at one month after the initial treatment of ranibizumab. The M-CHARTS score is the higher score of vertical and horizontal scores.

**Table 6:** Association between Posttreatment M-CHARTS Score and Other Baseline Measurement Values Associated with Acute Branch Retinal Vein Occlusion.

	r	p-value
Age	0.20	0.215
Visual acuity (logMAR)	0.29	0.061
Total foveal thickness	0.35	0.024
Thickness of serous retinal detachment	0.20	0.210
Maximum of total retinal thickness	0.16	0.318
Maximum of inner retinal thickness	0.11	0.494
Maximum of outer retinal thickness	0.25	0.114
Baseline M-CHARTS score	0.78	<0.001

logMAR, logarithm of the minimum angle of resolution.

The M-CHARTS score is the higher score of the vertical and horizontal scores.

## Discussion

In the current study, metamorphopsia associated with acute BRVO was quantified by M-CHARTS. Of 42 eyes, metamorphopsia was detected in 29 (69.0%) eyes. The mean vertical and horizontal scores were 0.59 ± 0.57 and 0.52 ± 0.67, respectively. Kinoshita et al.[20] reported that an M-CHARTS score of 0.3 to 0.5 may be the threshold for detecting patients with symptomatic metamorphopsia in their daily life. Twenty-five (59.5%) eyes in the current study had M-CHARTS scores equal to or lager than 0.5 at baseline (data not shown). We can estimate that approximately 60% of patients with acute BRVO have symptomatic metamorphopsia.

Previous reports showed that the severity of metamorphopsia due to epiretinal membrane is primarily related to the thickness of the inner nuclear layer [15, 21, 30]. With the use of an Amsler grid, Watanabe et al.[30] reported that metamorphopsia was detected in the area of edematous inner nuclear layer in eyes with epiretinal membrane. In our patients with BRVO, however, the M-CHARTS score had no correlation with the inner retinal thickness. Rather, the M-CHARTS score was correlated with the height of serous retinal detachment and the maximum outer retinal thickness. We can speculate that metamorphopsia from acute BRVO is mainly involved in the morphological changes of the outer retina.

One month after treatment with ranibizumab, the mean M-CHARTS score was slightly decreased. However, most eyes had persistent metamorphopsia in spite of the reduction of ME. Of 29 eyes that had metamorphopsia at baseline, only three eyes achieved complete resolution. In an analysis of 5 eyes with BRVO, Achiron et al.[5] reported that there was no improvement in the M-CHARTS score after the treatments. Similarly, Nakagawa et al.[6] reported that the M-CHARTS score was unchanged of 6 months in 12 eyes with acute BRVO. Metamorphopsia due to various diseases has been reported to be decreased as a result of treatment [5, 20, 24, 29]. However, metamorphopsia from BRVO tends to persist even after complete resolution of the ME.

Of 42 of our patients, 28 had metamorphopsia one month after the treatment. The posttreatment M-CHARTS score was not associated with any posttreatment factors. However, the posttreatment M-CHARTS score showed a close correlation with the baseline M-CHARTS score (r = 0.78, p < 0.001). Once metamorphopsia is induced by morphological changes of the retina caused by acute BRVO, this symptom often persists, even after the resolution of ME. In addition, two eyes developed metamorphopsia after the treatment. At baseline, metamorphopsia may not have been recognized in these patients due to the severe impairment of visual function [6].

To date, various pathophysiological mechanisms have been proposed for metamorphopsia [32–34]. Deformation of the foveal pit or uneven focal retinal thickening or the presence of cystoid spaces due to BRVO may cause metamorphopsia [32]. However, based on the limited improvement of metamorphopsia after the complete resolution of ME, these mechanisms could not explain metamorphopsia from acute BRVO. In eyes with epiretinal membrane [15, 21, 30], Okamoto et al.[15] speculated that the structural changes of horizontal cells, bipolar cells, amacrine cells, and Müller cells would inhibit the normal function of synaptic junctions and lower photoreceptor sensitivity, causing metamorphopsia.

In eyes with BRVO, Yamaike et al.[35] reported a correlation between VA and the integrity of the outer aspect of the foveal photoreceptor layer. Similarly, metamorphopsia from acute BRVO may be caused by the morphological changes of the outer aspect of the retina. In addition, the height of serous retinal detachment was correlated with the M-CHARTS score, and anteroposterior disorganization of the photoreceptor might be involved in metamorphopsia form acute BRVO [31]. Recently, adaptive optics-scanning laser ophthalmoscopy showed the decreased cone density and the disrupted cone mosaic arrangement in the parafoveal area in eyes with resolved BRVO [36]. Such disarray of the photoreceptors after the absorbance of ME may account for the persistent metamorphopsia in eyes with BRVO.

One of the major limitations of the current study is the small sample size. In addition, dense retinal hemorrhage from acute BRVO sometimes made it difficult to analyze the structural condition of the retina. In the current study, we evaluated the morphological changes of the retina using OCT, but it would be difficult to evaluate the disarray of each photoreceptor cell using this technique due to its relatively lower resolution in the retinal plane [37]. In addition, we aimed to elucidate the pathomorphology that caused the metamorphopsia in eyes with acute BRVO. We reevaluated the metamorphopsia one month after the treatment because most eyes showed complete reduction of ME at this time point [38]. We did not identify any factors that were predictive of the final prognosis of the visual symptoms.

Despite these shortcomings, we were able to quantitatively evaluate the metamorphopsia associated with acute BRVO using M-CHARTS. Even after the reduction of ME with anti-vascular endothelial growth factor agents, some patients still suffer from decreased quality of vision due to symptomatic metamorphopsia. Further prospective studies with longer follow-up periods are necessary to elucidate the long-term changes of metamorphopsia associated with BRVO.

## Supporting Information

S1 File Specific dataset for all individuals. (XLSX)

## References

[1] Hayreh SS. Ocular vascular occlusive disorders: natural history of visual outcome. Prog Retin Eye Res. 2014;41:1–25. Epub 2014/04/29. 10.1016/j.preteyeres.2014.04.001 S1350-9462(14)00025-1 [pii]. 24769221 PMC4073304

[2] Heier JS, Campochiaro PA, Yau L, Li Z, Saroj N, Rubio RG, et al Ranibizumab for macular edema due to retinal vein occlusions: long-term follow-up in the HORIZON trial. Ophthalmology. 2012;119(4):802–9. Epub 2012/02/04. 10.1016/j.ophtha.2011.12.005 S0161-6420(11)01151-1 [pii]. . 22301066

[3] Campochiaro PA, Clark WL, Boyer DS, Heier JS, Brown DM, Vitti R, et al Intravitreal aflibercept for macular edema following branch retinal vein occlusion: the 24-week results of the VIBRANT study. Ophthalmology. 2015;122(3):538–44. Epub 2014/10/16. 10.1016/j.ophtha.2014.08.031 S0161-6420(14)00790-8 [pii]. . 25315663

[4] Jonas J, Paques M, Mones J, Glacet-Bernard A. Retinal vein occlusions. Dev Ophthalmol. 2010;47:111–35. Epub 2010/08/13. 10.1159/000320076 000320076 [pii]. . 20703046

[5] Achiron A, Lagstein O, Glick M, Gur Z, Bartov E, Burgansky-Eliash Z. Quantifying metamorphopsia in patients with diabetic macular oedema and other macular abnormalities. Acta Ophthalmol. 2015 Epub 2015/04/23. 10.1111/aos.12735 . 25899144

[6] Nakagawa T, Harino S, Iwahashi Y. [Quantification of metamorphopsia in the course of branch retinal vein occlusion with M-CHARTS]. Nippon Ganka Gakkai Zasshi. 2007;111(4):331–5. Epub 2007/04/28. . 17461039

[7] Arimura E, Matsumoto C, Nomoto H, Hashimoto S, Takada S, Okuyama S, et al Correlations between M-CHARTS and PHP findings and subjective perception of metamorphopsia in patients with macular diseases. Invest Ophthalmol Vis Sci. 2011;52(1):128–35. Epub 2010/08/27. [pii]. . 20739469 10.1167/iovs.09-3535

[8] Faes L, Bodmer NS, Bachmann LM, Thiel MA, Schmid MK. Diagnostic accuracy of the Amsler grid and the preferential hyperacuity perimetry in the screening of patients with age-related macular degeneration: systematic review and meta-analysis. Eye (Lond). 2014;28(7):788–96. Epub 2014/05/03. [pii]. 24788016 10.1038/eye.2014.104 PMC4094801

[9] McGowan G, Yorston D, Strang NC, Manahilov V. D-CHART: A Novel Method of Measuring Metamorphopsia in Epiretinal Membrane and Macular Hole. Retina. 2015 Epub 2015/10/07. . 26441261 10.1097/IAE.0000000000000778

[10] Kim JW, Kim YT. Clinical application of 3D display device in ophthalmology: measurement of metamorphopsia. Acta Ophthalmol. 2015 Epub 2015/06/26. 10.1111/aos.12795 . 26109491

[11] Matsumoto C, Arimura E, Okuyama S, Takada S, Hashimoto S, Shimomura Y. Quantification of metamorphopsia in patients with epiretinal membranes. Invest Ophthalmol Vis Sci. 2003;44(9):4012–6. Epub 2003/08/27. . 12939323 10.1167/iovs.03-0117

[12] Arimura E, Matsumoto C, Okuyama S, Takada S, Hashimoto S, Shimomura Y. Retinal contraction and metamorphopsia scores in eyes with idiopathic epiretinal membrane. Invest Ophthalmol Vis Sci. 2005;46(8):2961–6. Epub 2005/07/27. 46/8/2961 [pii] 10.1167/iovs.04-1104 . 16043872

[13] Ooto S, Hangai M, Takayama K, Sakamoto A, Tsujikawa A, Oshima S, et al High-resolution imaging of the photoreceptor layer in epiretinal membrane using adaptive optics scanning laser ophthalmoscopy. Ophthalmology. 2011;118(5):873–81. Epub 2010/11/16. 10.1016/j.ophtha.2010.08.032 S0161-6420(10)00880-8 [pii]. . 21074858

[14] Kinoshita T, Imaizumi H, Okushiba U, Miyamoto H, Ogino T, Mitamura Y. Time course of changes in metamorphopsia, visual acuity, and OCT parameters after successful epiretinal membrane surgery. Invest Ophthalmol Vis Sci. 2012;53(7):3592–7. Epub 2012/05/17. [pii]. . 22589432 10.1167/iovs.12-9493

[15] Okamoto F, Sugiura Y, Okamoto Y, Hiraoka T, Oshika T. Associations between metamorphopsia and foveal microstructure in patients with epiretinal membrane. Invest Ophthalmol Vis Sci. 2012;53(11):6770–5. Epub 2012/09/13. [pii]. . 22969078 10.1167/iovs.12-9683

[16] Dell'omo R, Cifariello F, Dell'omo E, De Lena A, Di Iorio R, Filippelli M, et al Influence of retinal vessel printings on metamorphopsia and retinal architectural abnormalities in eyes with idiopathic macular epiretinal membrane. Invest Ophthalmol Vis Sci. 2013;54(12):7803–11. Epub 2013/11/10. 10.1167/iovs.13-12817 iovs.13-12817 [pii]. . 24204051

[17] Nomoto H, Matsumoto C, Arimura E, Okuyama S, Takada S, Hashimoto S, et al Quantification of changes in metamorphopsia and retinal contraction in eyes with spontaneous separation of idiopathic epiretinal membrane. Eye (Lond). 2013;27(8):924–30. Epub 2013/06/01. [pii]. 23722721 10.1038/eye.2013.108 PMC3740308

[18] Uji A, Murakami T, Unoki N, Ogino K, Nishijima K, Yoshitake S, et al Parallelism as a novel marker for structural integrity of retinal layers in optical coherence tomographic images in eyes with epiretinal membrane. Am J Ophthalmol. 2014;157(1):227–36 e4. Epub 2013/10/22. [pii]. . 24139623 10.1016/j.ajo.2013.09.008

[19] Kinoshita T, Imaizumi H, Miyamoto H, Katome T, Semba K, Mitamura Y. Two-year results of metamorphopsia, visual acuity, and optical coherence tomographic parameters after epiretinal membrane surgery. Graefes Arch Clin Exp Ophthalmol. 2015 Epub 2015/09/01. 10.1007/s00417-015-3147-3 . 26319984

[20] Kinoshita T, Imaizumi H, Miyamoto H, Okushiba U, Hayashi Y, Katome T, et al Changes in metamorphopsia in daily life after successful epiretinal membrane surgery and correlation with M-CHARTS score. Clin Ophthalmol. 2015;9:225–33. Epub 2015/02/14. 10.2147/OPTH.S76847 opth-9-225 [pii]. 25678770 PMC4322879

[21] Okamoto F, Sugiura Y, Okamoto Y, Hiraoka T, Oshika T. Inner nuclear layer thickness as a prognostic factor for metamorphopsia after epiretinal membrane surgery. Retina. 2015;35(10):2107–14. Epub 2015/05/16. . 25978729 10.1097/IAE.0000000000000602

[22] Okamoto F, Sugiura Y, Okamoto Y, Hiraoka T, Oshika T. Metamorphopsia and optical coherence tomography findings after rhegmatogenous retinal detachment surgery. Am J Ophthalmol. 2014;157(1):214–20 e1 Epub 2013/10/09. 10.1016/j.ajo.2013.08.007 S0002-9394(13)00544-8 [pii]. . 24099274

[23] Nowomiejska K, Oleszczuk A, Brzozowska A, Grzybowski A, Ksiazek K, Maciejewski R, et al M-charts as a tool for quantifying metamorphopsia in age-related macular degeneration treated with the bevacizumab injections. BMC Ophthalmol. 2013;13:13 Epub 2013/04/17. 10.1186/1471-2415-13-13 1471-2415-13-13 [pii]. 23587218 PMC3639210

[24] Krasnicki P, Dmuchowska DA, Pawluczuk B, Proniewska-Skretek E, Mariak Z. Metamorphopsia before and after full-thickness macular hole surgery. Adv Med Sci. 2015;60(1):162–6. Epub 2015/03/04. 10.1016/j.advms.2015.01.006 S1896-1126(15)00007-3 [pii]. . 25732531

[25] Okamoto Y, Okamoto F, Hiraoka T, Oshika T. Vision-related quality of life and visual function following intravitreal bevacizumab injection for persistent diabetic macular edema after vitrectomy. Jpn J Ophthalmol. 2014;58(4):369–74. Epub 2014/04/30. 10.1007/s10384-014-0323-7 . 24777841

[26] Arimura E, Matsumoto C, Okuyama S, Takada S, Hashimoto S, Shimomura Y. Quantification of metamorphopsia in a macular hole patient using M-CHARTS. Acta Ophthalmol Scand. 2007;85(1):55–9. Epub 2007/01/25. AOS729 [pii] 10.1111/j.1600-0420.2006.00729.x . 17244211

[27] Fukuda S, Okamoto F, Yuasa M, Kunikata T, Okamoto Y, Hiraoka T, et al Vision-related quality of life and visual function in patients undergoing vitrectomy, gas tamponade and cataract surgery for macular hole. Br J Ophthalmol. 2009;93(12):1595–9. Epub 2009/07/03. 10.1136/bjo.2008.155440 bjo.2008.155440 [pii]. . 19570766

[28] Bae SW, Chae JB. Assessment of metamorphopsia in patients with central serous chorioretinopathy. Indian J Ophthalmol. 2013;61(4):172–5. Epub 2013/05/21. 10.4103/0301-4738.112162 IndianJOphthalmol_2013_61_4_172_112162 [pii]. 23685489 PMC3714955

[29] Fujita K, Imamura Y, Shinoda K, Matsumoto CS, Mizutani Y, Mizota A, et al Quantification of metamorphopsia in chronic central serous chorioretinopathy after half-dose verteporfin photodynamic therapy. Retina. 2014;34(5):964–70. Epub 2014/01/11. . 24406387 10.1097/IAE.0000000000000027

[30] Watanabe A, Arimoto S, Nishi O. Correlation between metamorphopsia and epiretinal membrane optical coherence tomography findings. Ophthalmology. 2009;116(9):1788–93. Epub 2009/08/01. [pii]. . 19643494 10.1016/j.ophtha.2009.04.046

[31] Tsujikawa A, Sakamoto A, Ota M, Kotera Y, Oh H, Miyamoto K, et al Serous retinal detachment associated with retinal vein occlusion. Am J Ophthalmol. 2010;149(2):291–301 e5. Epub 2010/01/28. 10.1016/j.ajo.2009.09.007 S0002-9394(09)00673-4 [pii]. . 20103055

[32] Simunovic MP. Metamorphopsia and Its Quantification. Retina. 2015;35(7):1285–91. Epub 2015/06/08. . 26049620 10.1097/IAE.0000000000000581

[33] Midena E, Vujosevic S. Metamorphopsia: An Overlooked Visual Symptom. Ophthalmic Res. 2015;55(1):26–36. Epub 2015/11/12. [pii]. . 26554918 10.1159/000441033

[34] Wiecek E, Lashkari K, Dakin SC, Bex P. Novel quantitative assessment of metamorphopsia in maculopathy. Invest Ophthalmol Vis Sci. 2015;56(1):494–504. Epub 2014/11/20. [pii]. 25406293 10.1167/iovs.14-15394 PMC4299468

[35] Yamaike N, Tsujikawa A, Ota M, Sakamoto A, Kotera Y, Kita M, et al Three-dimensional imaging of cystoid macular edema in retinal vein occlusion. Ophthalmology. 2008;115(2):355–62 e2 Epub 2007/08/07. S0161-6420(07)00570-2 [pii] 10.1016/j.ophtha.2007.04.052 . 17675242

[36] Akagi-Kurashige Y, Tsujikawa A, Ooto S, Makiyama Y, Muraoka Y, Kumagai K, et al Retinal microstructural changes in eyes with resolved branch retinal vein occlusion: an adaptive optics scanning laser ophthalmoscopy study. Am J Ophthalmol. 2014;157(6):1239–49 e3 Epub 2014/02/18. [pii]. . 24531026 10.1016/j.ajo.2014.02.026

[37] Stanga PE, Bird AC. Optical coherence tomography (OCT): principles of operation, technology, indications in vitreoretinal imaging and interpretation of results. Int Ophthalmol. 2001;23(4–6):191–7. Epub 2002/04/12. . 11944840 10.1023/a:1014476430078

[38] Ota M, Tsujikawa A, Miyamoto K, Sakamoto A, Murakami T, Yoshimura N. Visual acuity following intravitreal bevacizumab for macular edema associated with retinal vein occlusion. Jpn J Ophthalmol. 2010;54(6):555–64. Epub 2010/12/31. 10.1007/s10384-010-0878-x . 21191716

